# Fern-like Plants Establishing the Understory of the Late Devonian Xinhang Lycopsid Forest

**DOI:** 10.3390/life14050602

**Published:** 2024-05-08

**Authors:** Jiangnan Yang, Deming Wang, Le Liu, Yi Zhou

**Affiliations:** 1 Key Laboratory of Orogenic Belts and Crustal Evolution, Department of Geology, Peking University, Beijing 100871, China; 2 School of Geoscience and Surveying Engineering, China University of Mining and Technology (Beijing), Beijing 100083, China; 3 School of Life Sciences, Sun Yat-sen University, Guangzhou 510275, China

**Keywords:** procumbent stems, roots, fern-like plants, *Xinhangia*, Xinhang forest, Late Devonian

## Abstract

Forests appeared during the Middle to Late Devonian, but Devonian forests and their compositions are still rarely known. Xinhang forest was reported as the largest Devonian forest, with lycopsid trees of *Guangdedendron micrum* Wang et al. A fern-like plant *Xinhangia spina* Yang and Wang with shoots and anatomy, was previously described from this forest, but its habit and ecology remain unclear. From Xinhang forest, we now report more specimens of fern-like plants including *X. spina* and some unnamed plants in several beds. Prominent adventitious roots, spines and secondary xylem indicate that the stems of *X. spina* are largely procumbent to function as anchorage, absorption and support. Other fern-like plants with distinct roots or multiple slender branches also suggest procumbent habits. Xinhang forest is thus reconsidered as multispecific with a canopy of lycopsid trees and understory of diverse fern-like plants, which are adapted to the disturbed coastal environment. The composition of Xinhang forest may indicate a structural transition of the early forests’ dominator from fern-like plants to lycopsids.

## 1. Introduction

Devonian is a key evolutionary period for tracheophytes (vascular plants), including lycopsids, sphenopsids, seed plants and fern-like plants, and they all underwent great development, differentiation and diversity during this time [1]. Among them, fern-like plants extending from the Middle Devonian to Carboniferous are composed of iridopteridaleans, pseudosporochnaleans, non-pseudosporochnaleans, rhacophytaleans and stauropteridaleans [2,3]. They demonstrate abundant morphological and anatomical characters and show complex phylogenetic relationships [4,5,6], but their living niches are poorly known.

The emergence of forests profoundly remodeled the terrestrial ecosystem and global environment [7,8,9,10]. As for the earliest forests in the Devonian, the Middle Devonian (Givetian) Cario and Gilboa forests in New York, NY, USA, are dominated by the arboreous (trees of) fern-like plant *Eospermatopteris* and progymnosperms including *Archaeopteris* and/or aneurophytalean taxa [11,12]. Of the two Late Devonian forests mainly consisting of trees of lycopsids, one is the Frasnian Svalbard forest from Norway [13] and the other is the Famennian Xinhang forest from China [14,15].

Xinhang forest has thousands of in-situ lycopsid trees of *Guangdedendron micrum* and is distributed over an area of over 25 hectares. With continual excavation, new fossil plants other than lycopsids were recently discovered from this forest. Among them, *Xinhangia spina* is a fern-like plant characterized by an aerial stem bearing alternate to triseriate primary branches, simple ultimate appendages and clepsydroid-shaped stele [16]. By studying more specimens, this article focuses on *X. spina* with procumbent stems and other fern-like plants in Xinhang forest, whose habits are discussed in relation to the palaeoenvironment.

## 2. Material and Methods

**Figure 1:**
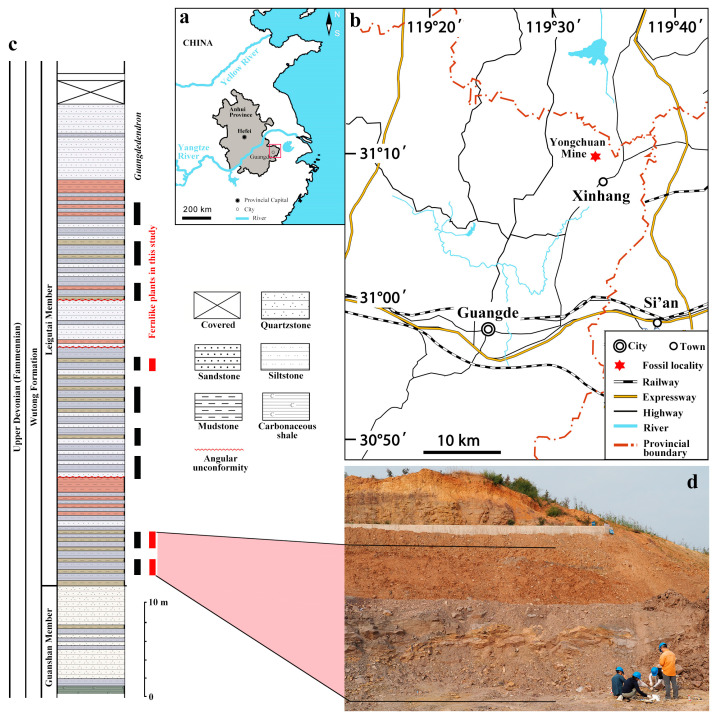
Location, stratigraphy of section and outcrop view. (**a**,**b**) Locality of the Yongchuan mine in Xinhang Town, Guangde City, Anhui Province, China. (**c**) Stratigraphic column of the Leigutai Member (Upper Devonian Wutong Formation) at the Yongchuan mine, showing the lithology and occurrence of *Guangdedendron* (black vertical lines) and the fern-like plants (red vertical lines) in this study. (**d**) Part view of outcrops at the Yongchuan mine, in which *Xinhangia* and some other plant fossils were found, showing our group members collecting the plant specimens beside the section.

**Figure 2:**
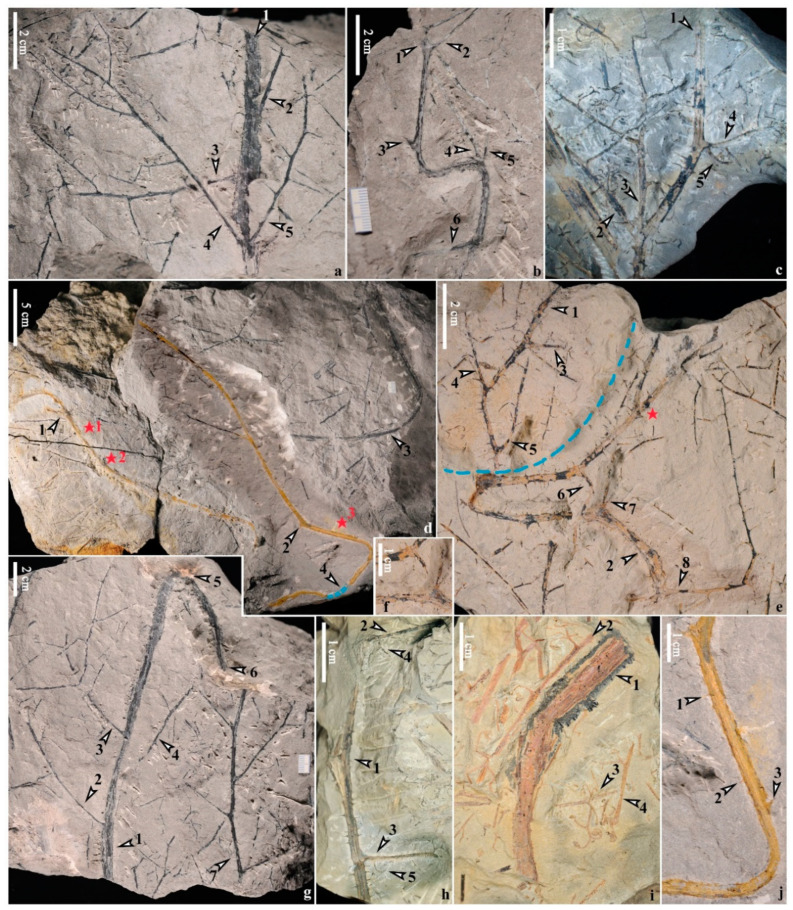
*Xinhangia spina* from the Yongchuan mine. (**a**) Straight stem (arrow 1) with alternately arranged (arrows 2, 3) or paired (arrows 4, 5) primary branches bearing alternate secondary branches. (**b**) Winding stem (arrow 1) bearing alternate (arrows 2, 3, 6) and paired (arrows 4, 5) primary branches. (**c**) Stem (arrow 1) with a pair of (arrows 2, 3) and single (arrow 4) primary branches arranged in a triseriate pattern. Arrow 5 indicating a dichotomous aphlebia at base of a primary branch (from Figure 1F in [16]). (**d**) Three long winding stems (arrows 1–3) bearing primary branches or branch bases, with the left stem (arrow 1) partially mineralized. Arrows 1 and 2 also indicating bifurcations of stems. Blue dotted line (arrow 4) suggests connection to stem. Red stars 1 and 2 indicating positions of xylem transverse sections in Figure 5h,i. Star 3 indicating enlargement in (**j**). (**e**) Two disconnected winding stems (arrows 1 and 2, separated by blue dotted line) bearing primary branches (arrows 3–8) and dense spines. Red star indicating a bifurcation of stem. (**f**) Exposed stem in **e** after removing overlying rock matrix (arrow 6). (**g**) Winding stem (arrow 1) bearing three primary branches (arrows 2–4). Arrow 5 indicating bending position. Arrow 6 indicating position where stem plugs into the rock matrix. Arrow 7 indicating a primary branch with alternate secondary branches. (**h**) Dichotomous stem (arrow 1) bearing two primary branches (arrows 2, 3). Arrows 4 and 5 indicating aphlebiae at the base of primary branches. (**i**) A spiny stem (arrow 1) surrounded by several fertile branches (arrows 2–4). (**j**) Enlarged stem in (**d**) (red star 3) showing superficial spines (arrows 1–3).

**Figure 3:**
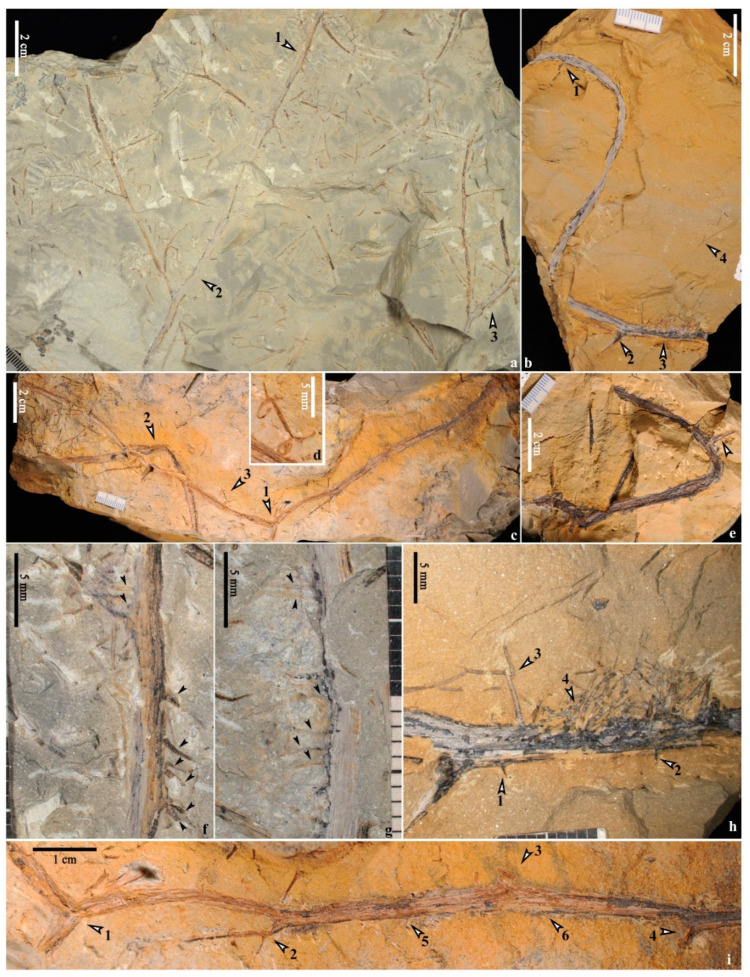
Procumbent and spiny stems of *Xinhangia spina* from the Yongchuan mine. (**a**) Three stems (arrows 1–3) with adventitious roots and primary branches bearing secondary branches arranged alternately. (**b**) Winding stem bearing primary branch bases (arrows 1 and 2) and adventitious roots on one side of stem (arrow 3). Arrow 4 indicating a disconnected root with bifurcation. (**c**) A long stem with a bifurcation (arrow 1) and alternate primary branch bases (enlarged in **i**). Another stem (arrow 2) and fertile organ (arrow 3, enlarged in (**d**)) in association. (**d**) Enlargement of the fertile organ in (**c**) (arrow 3). (**e**) Winding stem bearing a primary branch base (arrow). (**f**,**g**) Enlarged parts in (**a**) (arrows 1 and 2, respectively), showing adventitious roots on stems (small black arrows). (**h**) Enlarged part in (**b**) (arrow 3), showing adventitious roots on one side of stem with spines (arrows 1, 2). Arrows 3 and 4 indicating bifurcating roots. (**i**) Enlargement of the right part of stem in (**c**), showing stem bifurcation (arrow 1) and three primary branches or their bases (arrows 2–4). Arrows 5 and 6 indicating spines on stem.

**Figure 4:**
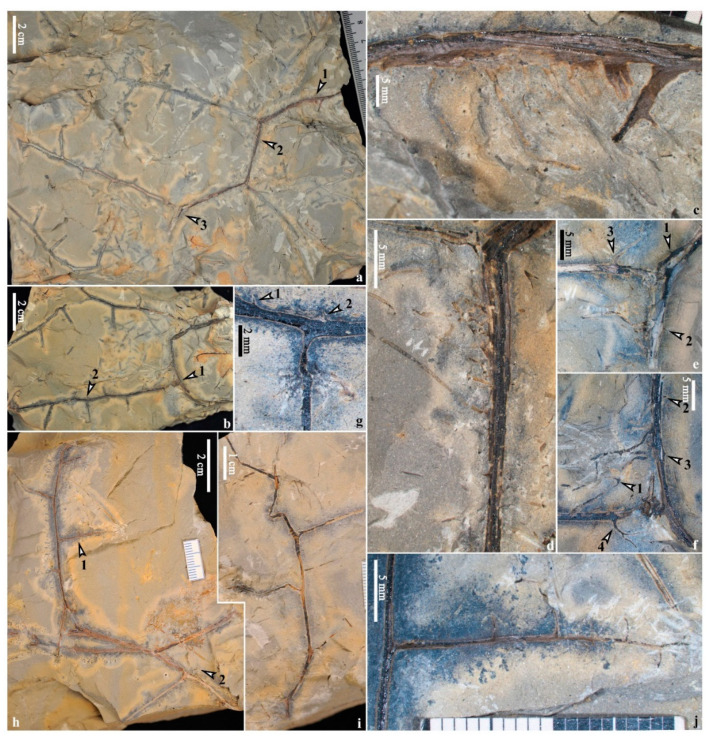
Procumbent stems of *Xinhangia spina* from the Yongchuan mine. (**a**,**b**) Part and counterpart of flexed stem with adventitious roots on one side of internodes, bearing primary branches on the nodes, with secondary branches alternately arranged. (**c**–**f**) Enlarged parts in (**a**,**b**) (arrows 1–3 in (**a**), arrow 1 in (**b**), respectively), showing details of adventitious roots (with occasional bifurcation, arrow 1 in (**f**)) arranged on one side of stem, dichotomous aphlebia at base of primary branch (arrow 3 in (**e**), arrow 4 in (**f**)) and spines (arrows 1 and 2 in (**e**), arrows 2 and 3 in (**f**)). (**g**) Enlarged part in (**b**) (arrow 2), showing primary branch (the wider axis) with spines (arrows 1–2), bearing a secondary branch (the thinner axis). (**h**) Primary branch with secondary branches arranged alternately. Arrow 1 indicating enlargement in (**j**). Arrow 2 indicates an aphlebia at the base of a primary branch. (**i**) Primary branch with secondary branches arranged alternately. (**j**) Enlarged part in (**h**) (arrow 1), showing the whole secondary branch with hook-like vegetative ultimate appendages borne alternately.

**Figure 5:**
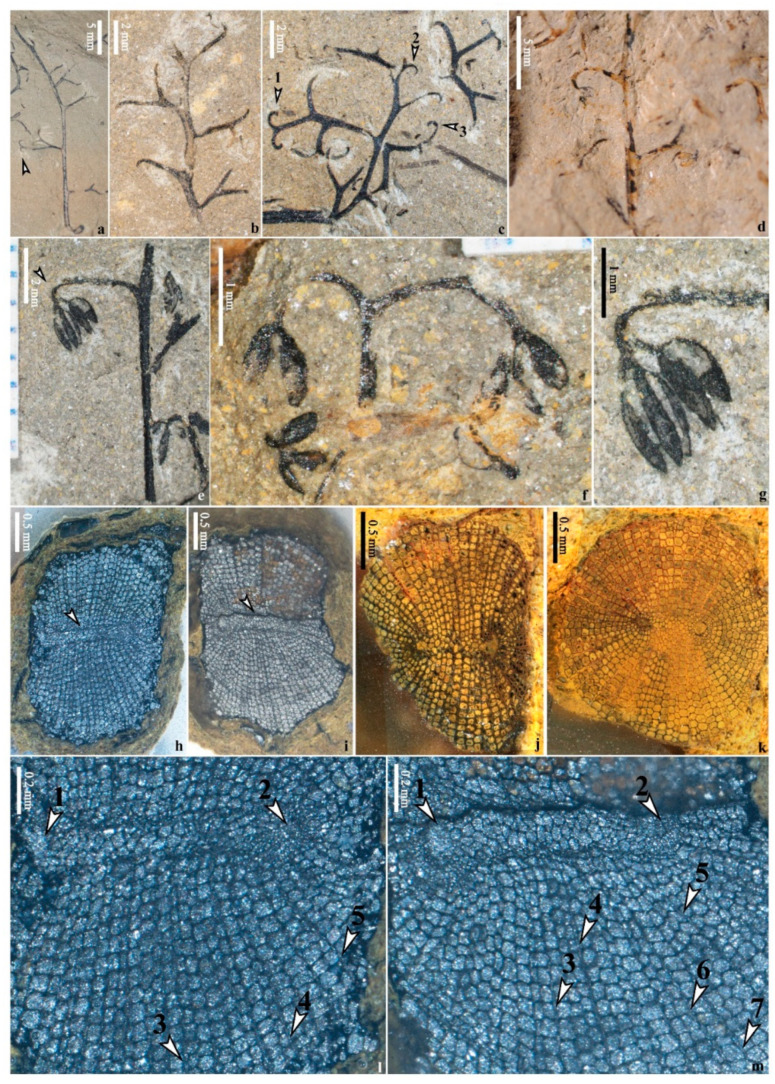
Ultimate appendages and anatomy of *Xinhangia spina* from the Yongchuan mine. (**a**–**c**) Vegetative branches alternating by dichotomous ultimate appendages with recurved tips. Arrows indicating such ultimate appendages ((**a**) from Figure 3A in [16])**.** (**d**) Fertile secondary branch bearing alternate fertile organs with terminal sporangia in pairs (from Figure 4M in [16]). (**e**) Fertile secondary branch bearing one fertile organ (arrow). (**f**) Fertile organ dichotomizing into two clusters of sporangia. (**g**) Enlarged fertile organ in (**e**) (arrow), which dichotomizes twice and terminates in paired and elongate sporangia. (**h**,**i**) Transverse sections of procumbent stem in Figure 2d (at two positions labelled by red star 1 and 2, respectively), showing elongate clepsydroid-shaped primary xylem (arrow) surrounded by radially arrayed secondary xylem. (**j**,**k**) Transverse sections of *Xinhangia* from Figure 7G,K in [16], respectively, showing clepsydroid stele. (**l**,**m**) Enlarged parts in (**h**,**i**), respectively, showing details of primary and secondary xylem. Arrows 1 and 2 in (**l**,**m**) indicating protoxylem poles. Arrows 3–5 in (**l**) and 3–7 in (**m**) indicating rays.

**Figure 6:**
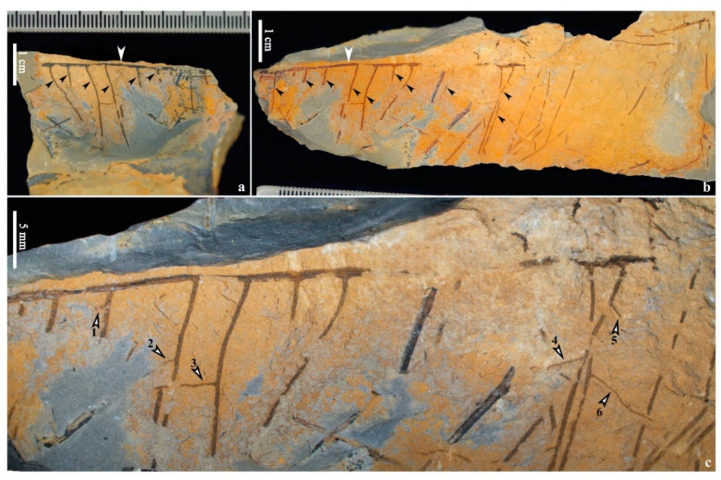
Roots of fern-like plant from the Yongchuan mine. (**a**,**b**) Part and counterpart of roots. White arrows indicating rhizome and black arrows indicate primary roots. (**c**) Enlargement of left part in (**b**), showing details of roots. Arrows 1–6 indicating lateral roots.

The specimens were obtained from the Leigutai Member (upper part of the Upper Devonian Wutong Formation), at Yongchuan clay mine, which is located in Xinhang Town, Guangde City, Anhui Province, China (Figure 1a,b). The fern-like plants in this study were preserved mainly within mudstone and siltstone, and distributed in both the bottom and the mid-upper beds of Leigutai Member (Figure 1c, vertical red lines), while in-situ roots and stems of lycopsid *Guangdedendron micrum* are found in almost the whole Leigutai Member (Figure 1c, vertical black lines). Most specimens (Figure 2, Figure 3, Figure 4, Figure 5, Figure 6, Figures S1 and S2) were obtained from the two lowest beds of this member (Figure 1c, the two lowest red lines; Figure 1d), which are separated by a thin bed of quartz sandstone. The others (Figure S3) are from the upper bed (Figure 1c, the top red line). The lycopsid *Sublepidodendron grabaui* Wang and Xu was found near the lower fossil-bearing bed of the Leigutai Member and has been described by Xu et al. [17]. 

Steel needles were used to expose the plant morphology. Embedding and polishing techniques were adopted to acquire anatomical information from the permineralized stems. A digital camera and a light microscope were manipulated to take the photographs and Adobe Photoshop CC2018 was used to prepare the figures. All the specimens studied are kept at the Department of Geology, Peking University, Beijing, China.

## 3. Systematics

Class and Order: *Incertae sedis*.

Genus: *Xinhangia* Yang and Wang 2022 emend.

Emended generic diagnosis (with emended and additional diagnoses in brackets):

Stems dichotomous [upright or procumbent, with adventitious roots arranged on one side]. Primary and secondary branches borne alternately and sometimes in a triseriate pattern. Vegetative or fertile ultimate appendages alternately arranged on secondary branches [or sometimes on primary branches]. Vegetative ultimate appendages with recurved tips [usually dichotomizing 0–2] times. Fertile ultimate appendages (= fertile organs) usually dichotomizing 1–2 times to terminate in elongated and paired sporangia. Primary xylem of [upright and procumbent] stems mesarch, clepsydroid-shaped with two protoxylem poles, and surrounded by secondary xylem. Secondary xylem rays uniseriate. Tracheid wall with scalariform thickenings or circular to elliptical bordered pits.

Type species: *Xinhangia spina* Yang and Wang 2022 emend.

Specific diagnosis (with emended and additional diagnoses in brackets):

As in the generic diagnosis. Stems [1.8–12.6 mm] wide and up to [35 cm] long, with primary branches arranged at 45–90°. [Adventitious roots on stems 0.3–0.6 mm wide and up to 1.3 cm long, with occasional bifurcation]. Primary branches 0.7–3.0 mm wide and up to [9.8 cm] long; secondary branches [0.3–1.2 mm] wide and up to [5.8 cm] long; tertiary branches 0.2–0.4 mm wide and up to 7.0 mm long. Sometimes a dichotomous aphlebia is inserted at the base of primary and secondary branches. Spines on stems and primary branches, [0.5–3.0 mm] long. Basal axes within fertile ultimate appendages 0.2–0.4 mm wide. Sporangia 0.3–0.5 mm wide and 0.9–1.5 mm long. Xylem column ca. 2.0 mm in diameter in stems. Primary xylem [(205–360) μm × (720–1740) μm] in transverse section. Tracheids in protoxylem, metaxylem and secondary xylem [10–31 μm, 30–75 μm, 32–108 μm] in diameter, respectively.

## 4. Results

### 4.1. Xinhangia spina

#### 4.1.1. Upright and Procumbent Stems

Numerous specimens show spiny stems (Figure 2, Figure 3, Figure 4 and Figure S1, st). A few stems are straight (Figure 2a,h and Figure 3a), while others are winding or curved (Figure 2b–g,i and Figure 3b,c,e) or zigzags (Figure 4a,b). Upright stems (Figure 2c,h,i) are 1.8–(3.3, n = 3)–5.0 mm wide and up to 9.2 cm long (mean value, n = number of measurements in parentheses, similarly hereinafter). Procumbent stems (Figure 2b,d–g, Figure 3a–e and Figure 4a,b,h) are 2.0–(3.6, n = 24)–5.5 mm wide in most cases but could be 9.5 mm wide in one specimen (Figure 2a, arrow 1). The longest procumbent stem could reach ca. 35 cm if the missing part (Figure 2d, arrow 4, blue dashes) is added. Bifurcations occur in both upright (Figure 2h, arrow 1) and procumbent stems (Figure 2d, arrows 1,2; Figure 2e, red star; Figure 2h, arrow 1; Figure 3c, arrow 1). 

Four specimens show stems with adventitious roots (Figure 3a,b and Figure 4a,b). Usually, adventitious roots are arranged one side (Figure 3g, arrows; Figure 3h, Figure 4c–f and Figure S1d,e,g, ar) of the stems. These roots are 0.3–(0.4, n = 18)–0.6 mm wide and up to 1.3 cm long and bifurcate occasionally (Figure 3b, arrow 4; Figure 3h, arrows 3,4; Figure 4f, arrow 1). An unusual case shows that the roots seem to be arranged on both sides of the stem (Figure 3f, arrows), but it could not be the original state due to the preservation. Spines could be clearly observed on both upright and procumbent stems (e.g., Figure 2c,e,h and Figure 3h, arrows 1,2; Figure 3i, arrows 5,6; Figure 4e, arrows 1,2; Figure 4f, arrows 2,3) and even the permineralized ones (e.g., Figure 2j, arrows 1–3). 

#### 4.1.2. Primary and Secondary Branches

On both the upright and procumbent stems, the primary branches measure 0.5–(1.1, n = 21)–2.0 mm wide and up to 9.8 cm long (mean value, n = number of measurements in parentheses, similarly hereinafter), arranged alternately in most cases (Figure 2a, arrows 2,3; Figure 2b, arrows 2,3,6; Figure 2e, arrows 3–8; Figure 3i, arrows 2–4, with only bases preserved; Figure 4a) but sometimes in a triseriate pattern (paired branches: Figure 2a, arrows 4,5; Figure 2b, arrows 4,5; Figure 2c, arrows 2,3); sometimes, a single dichotomous aphlebia is arranged at the base of the primary branches (Figure 2c, arrow 5; Figure 2h, arrows 4,5; Figure 4e, arrow 3; Figure 4f, arrow 4; Figure 4h, arrow 2). In most cases, the secondary branches are alternately arranged on the primary branches (Figure 2a, arrows 3–5; Figure 2c, arrows 2,3; Figure 2g, arrow 7; Figure 3a, Figure 4a,b and Figure S1a,d,g, sb), measuring 0.3–(0.7, n = 12)–1.2 mm wide and up to 5.8 cm long, and bear vegetative ultimate appendages in alternate arrangement (Figure 4j and Figure 5a,b). Sometimes, such appendages are inserted on the primary branches directly (Figure 2e and Figure S1c, vua). When the primary and secondary branches bear vegetative ultimate appendages, the branches are spineless (e.g., Figure 2e, arrow 8; Figure 5a–c). 

#### 4.1.3. Vegetative Ultimate Appendages and Fertile Organs

Vegetative ultimate appendages usually dichotomize once or twice with recurved tips (Figure 5a–c) but sometimes do not dichotomize on the primary (Figure 2e and Figure S1c, vua) or secondary (Figure 4j) branches. Fertile organs are similar to the vegetative ultimate appendages with dichotomy and recurving but bear terminal elongate sporangia in pairs (Figure 2i, arrows 2,3; Figure 5d–g). 

#### 4.1.4. Anatomy

One pyritized procumbent stem containing only the xylem was embedded and transversely cut into tens of sections (Figure 2d, arrow 1). Two representative slices show the anatomical traits (Figure 5h,i). The stele is ca. 2.0 mm in diameter and consists of a primary xylem surrounded by secondary xylem. The primary xylem is mesarch and clepsydroid-shaped in cross section (Figure 5h,i, arrow), measuring 1138–(1383, n = 13)–1740 μm by 205–(279, n = 13)–348 μm (mean value, n = number of measurements in parentheses, similarly hereinafter). At two ends of primary xylem, there is a distinct protoxylem pole, respectively (Figure 5l,m, arrows 1,2). The small tracheids around the two poles, measuring 10–(16, n = 28)–31 μm in diameter, indicate protoxylem, and the larger tracheids between the poles and around the protoxylem represent metaxylem and measure 31–(43, n = 28)–75 μm in diameter. The radially arrayed tracheids around the primary xylem belong to secondary xylem and measure 43–(69, n = 33)–108 μm in diameter (Figure 5l,m). Abundant rays can be identified among the secondary xylem tracheids (Figure 5l, arrows 3–5; Figure 5m, arrows 3–7).

### 4.2. Other Fern-like Plants

A pair of specimens with only the rooting system preserved along the bedding plane (Figure 6a–c) shows the possible original state when living. The long straight rhizome (Figure 6a,b, white arrows) is 0.6–(0.7, n = 4)–0.9 mm wide and up to 6.7 cm long (mean value, n = number of measurements in parentheses, similarly hereinafter). Primary roots occurring on one side of the rhizome are parallel and 0.2–(0.4, n = 16)–0.5 mm wide constantly and up to 25 mm long (Figure 6a,b, black arrows). The intervals between adjacent primary roots are 3.7–6.5 mm. Secondary roots are occasionally visible and 0.1–0.2 mm (n = 6) wide and up to 6.5 mm long (Figure 6c, arrows 1–6). 

Some fern-like plants are associated with *Guangdedendron micrum* at the bottom of the Leigutai Member (Figure 1c, the two lowest vertical red lines; Figure S2a–l). Several in-situ trunks (Figure S2d–f, arrows) and erect strobili (Figure S2g, arrows) of *G. micrum* occur on the highwall. Debris of fern-like plants (Figure S2i,j, arrows) and strobili (Figure S2k, arrow) of *G. micrum* were preserved together. A relatively well-preserved fern-like plant shows several adjacent stems, 1.5–4.0 mm (n = 4) wide and up to 11 cm long (Figure S2l, arrows 1–3), bearing possible adventitious roots (Figure S2l, arrow 4) and primary branches. 

Fern-like plants are also found at a higher bed in the Leigutai Member (Figure 1c, the top red line; Figure S3a–c). Their numerous branches are preserved with the trunks of *Guangdedendron micrum* (Figure S3a–c). These branches are 0.8–(1.6, n = 17)–2.3 mm wide and over 12 cm long and appear to be perpendicular to the tree trunks.

## 5. Discussion

### 5.1. Assignment of the Fern-like Plants from Xinhang Forest

**Figure 7:**
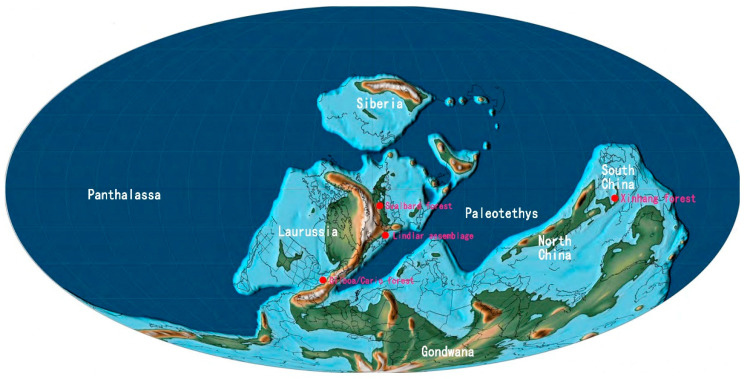
Distribution of the Devonian forests in the palaeomap (modified from [50]).

As previously studied, *Xinhangia spina* possesses characters including primary and secondary branches arranged in alternate and sometimes triseriate patterns, dichotomous vegetative and fertile ultimate appendages with rare divisions, and stele in a clepsydroid shape [16]. The current plants have characteristics including (1) procumbent, spiny, long and sometimes bifurcated stems, (2) primary branches arranged in alternate and sometimes triseriate pattern (Figure 2a–c,e,g), (3) single dichotomized aphlebia inserted at the bases of primary branches (Figure 4e, arrow 3; Figure 4f, arrow 4; Figure 4h, arrow 2), (4) secondary branches borne alternately (Figure 2a, arrows 3–5; Figure 2g, arrow 7), (5) dichotomous vegetative ultimate appendages with recurved tips (Figure 5a–c), (6) dichotomous fertile organs terminating in paired sporangia (Figure 5d–g), and (7) clepsydroid-shaped primary xylem surrounded by secondary xylem (Figure 5h,i). These characters fit *X*. *spina* well and expand its diagnoses in procumbent stems, ultimate appendages sometimes born directly on primary branches and assignment of xylem column to stems. 

The root in Figure 6 is tentatively allied to fern-like plants because it differs from the roots typical of the lycopsids, sphenopsids and progymnosperms. The cormose and stigimarian roots of the lycopsids show a swollen base and branched rhizomorph axes, respectively [14]. As for Devonian–Permian sphenophyllalean sphenopsids, the adventitious root clusters occur on nodes of the creeping axes (Liu Le et al., in preparation). Relating to the progymnosperms, *Archaeopteris* shows numerous primary roots diverging from the bases of a single central trunk [12]. With regard to the aneurophytalean progymnosperms, unbranched roots envelop the straight or curved horizontal rhizomes [11]. The roots of Devonian seed plants are currently unknown; thus, a comparison here is impossible. The seeds, if present in Yongchuan mine, are mostly isolated. Therefore, it is unlikely that the root in Figure 6 relates to the seed plants. The plants with multiple branches in Figures S2 and S3 are assigned to fern-like plants generally because they lack organs, including the following: (1) microphylls and strobili, (2) nodes and internodes, (3) pinnate sporangia and/or megaphylls, (4) seeds and pollen organs. These four groups of vegetative and/or fertile organs characterize the lycopsids, sphenopsids, progymnosperms and seed plants, respectively.

### 5.2. Habits of the Fern-like Plants from Xinhang Forest

Among Devonian fern-like plants, the order Pseudosporochnales contains some tree-sized members (e.g., *Pseudosporochnus*, *Lorophyton*, *Pietzschia* and *Eospermatopteris*) and contributes to the earliest forest ecosystems in the Devonian [11,12,18,19], while other fern-like plants are smaller in size and rarely known in relation to the forests [2]. In order to clarify the habits of Devonian fern-like plants including *Xinhangia*, detailed comparisons of relative morphology are given in Table 1. 

Quite a few Devonian fern-like plants with roots have been studied in detail (Table 1). Pseudosporochnaleans have a tree-like appearance and their roots are radially arranged around the base of the trunk, like the extant tree ferns or palms [11,18,33], which show great differences from the roots in this study. The aerial roots of *Denglongia* [27] and *Metacladophyton* [22] occur below some nodes of stems, while the roots of *Xinhangia* are born on internodes. *Xinhangia*’s roots are similar to those of *Shougangia* [3], *Rhacophyton* [24,25], *Melvillipteris* [27], *Ellesmeris* [28] and *Protoperidophyton* [29], and they are all inserted on the horizontal stems. Similarly, *Flabellopteris* [30] is interpreted with an erect habit connected to a horizontal rhizome, though evidence of roots is deficient. In the previous study, procumbent stems and roots are undetected in *Xinhangia* [16]. In this study, slender root systems are attached to stems (Figure 3a,b and Figure 4a,b) or found isolated (Figure 6), and some of them have bifurcation (Figure 3b, arrow 4; Figure 3h, arrows 3,4; Figure 4f, arrow 1) or one order of lateral roots (Figure 6c, arrows 1–6), which is rare among Devonian fern-like plants. The roots occur on one side of the stems (Figure 3a,b and Figure 4a,b) or rhizomes (Figure 6) in most cases, indicating that these fern-like plants are, at least partly, procumbent on the ground. The fern-like plants associated with the tree lycopsid *Guangdedendron* (Figures S2 and S3) are slender and multiply branched, indicating that they may also be procumbent in habit. In the face of the poor drainage and disturbed environment in Xinhang forest, the adventitious roots and well-developed secondary xylem of the stems may together help enhance the absorption of nutrients and/or physical stability in the substance. 

In addition to *Xinhangia*, spines were reported from most iridopteridaleans (e.g., *Anapaulia moodyi* Berry and Edwards [34], *Ibyka amphikoma* Skog and Banks [35]) and some other fern-like plants (e.g., *Metacladophyton ziguinum* Wang and Lin [21]; *Tsaia denticulate* Wang and Berry [36]), while not in pseudosporochnaleans and rhachophytaleans. In *Xinhangia spina*, spines are usually observed on the stems and primary branches but are rare on either vegetative or fertile ultimate branches in both previous and present studies. In contrast, some fern-like plants bear spines on ultimate branches (e.g., *Anapaulia moodyi*, *Ibyka amphikoma*, *Tsaia denticulate*), but reasons for their existence are little discussed. The spines of vascular plants are supposed to function as secretory and possible conducting tissue [37], assisting with clinging–climbing [38] or defensing against herbivores, especially for later vertebrates [39,40]. For some fern-like plants showing similar habits to *Xinhangia*, e.g., *Denglongia*, *Ellesmeris*, *Protopteridophyton* and *Shougangia*, they all lack spines, while their living environments are not known. Considering the disturbed environment to which Xinhang forest adapted [14], we suggest that the spines of *Xinhangia* may serve as anchors and graspers to help to firmly scramble on and fix to the ground or become tangled with each other for more support. Spines are widespread on procumbent stems with adventitious roots (e.g., Figure 3h and Figure 4e,f), suggesting that these two structures may function together as anchorages to defend the possible periodic flood. The primary branches bearing secondary branches or ultimate appendages grow uprightly for receiving light and spreading spores. When rising up, the plant does not need to grasp the ground, which could explain why the higher orders of branches and ultimate appendages lack spines.

### 5.3. Habitat and Environment 

Until now, only a few Devonian forests have been found and studied in detail (Table 2), and they all come from the Laurussia palaeocontinent except for Xinhang forest, in South China (Figure 7). Recently, a Famennian plant assemblage comprising lycopsids, fern-like plants and seed plants was also found from Gondwana palaeocontinent [41]. Forests can date back to the Middle Devonian, when pseudosporochnalean trees began to flourish. Eifelian Lindlar assemblage, Givetian Cairo and Gilboa forests from N.Y., USA, [9,12,13,18,42,43] are mainly composed of pseudosporochnalean trees, despite the fact that they are inferred to represent different living environments (Table 2). Just recently, a new study reports the earliest forest in England, four million years older than the one in Gilboa, which is also composed of pseudosporochnalean trees [19]. In the Late Devonian, structural transition in forests happened according to the existing evidence. The pseudosporochnaleans seemed to retreat from the dominant composition of forests, and instead, lycopsids took place of the main roles of forests in Svalbard of Norway and in Xinhang of China, especially the Carboniferous forests in Euramerica [13,14,44,45,46]. Since the Late Devonian, medium- to small-sized fern-like plants or ferns existed as bushy understory in swamps or mires, i.e., peat- and non-peat-forming wetlands [46]. Among them, Late Devonian *Rhacophyton* dominated the floodplain swamps and the understory in the shade of *Archaeopteris* or lycopsids [26,47,48,49], possibly similar to the fern-like plants in Xinhang forest.

The fern-like plants in this research were found from several beds in the Leigutai Member (Figure 1c), suggesting their existence for a long time. Materials from the previous study only contain shoot parts, and elaborate structures such as complete fertile organs attached to branches are preserved [16]. Thus, *Xinhangia* in both previous and present studies, as well as some other fern-like plants (Figure 6 and Figure S2) from the same bed (Figure 1c, the two lowest vertical red lines), are considered as being autochthonous or parautochthonous for the preservation of roots and/or well-preserved aerial branches. The fern-like plants could spread in the lycopsid forest as a possible understory vegetation, since they are often closely associated with the lycopsid trees of *Guangdedendron micrum* (Figures S2 and S3). These trees without evident canopies permitted the understory plants to receive sunlight. Thus, the vertical stratification of Xinhang forest could be built. The trees of *G. micrum* grew in a littoral habitat and were prone to be submerged by the frequent coastal flooding [14]. *Xinhangia* is considered as a primitive taxon with simple vegetative and fertile organs [16], which could be related to the disturbed environment for faster growth. As the biggest Devonian forest in the world, Xinhang forest may represent a universal forest structure at that time. Such combination of lycopsids and fern-like plants also seems to be a rudiment of the lycopsid–fern system in the Carboniferous forests [45,51].

The fossil bearing lens consists of mudstone and siltstone, indicating an environment with low energy. *Xinhangia* in both previous and present studies, as well as the roots in Figure 6, were not associated with the lycopsid trees of *Guangdedendron micrum*, suggesting that these fern-like plants might have survived in patches. These patches with low energy represented local habitats segregated by numerous lycopsid trees, which were adapted to tidal turbulent environments reflected by quartz sandstones, siltstones and mudstones. 

## Figures and Tables

**Table 1:** Comparisons of *Xinhangia* with related Devonian fern-like plants.

Taxon [References]	Age	Ultimate Appendages	Roots		Spines	Habits	Habitats	Schematic Drawing
			Types	Description				
*Xinhangia* [16], this study	Famennian	Dichotomous with recurved tips	Adventitious roots	On one side of procumbent stems with occasional bifurcation	On stems and 1st branches	Stem upright or procumbent with upright 1st branches	Living as understories in lycopsid forest near the coast	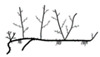
Pseudosporochnaleans [11,18]	Eifelian to Frasnian	Dichotomous and three dimensional	Roots	Radially arranged at base of trunk	Not observed	Tree size in shape, consisting of an upright trunk with densely inserted branches	Main components of forests in Middle Devonian	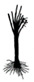
*Denglongia* [20]	Frasnian	Dichotomous, planate or three-dimensional	Aerial roots	On basal nodes of stems or nodes of 1st branches	Not observed	Herbaceous in growth habit or a subtree with secondary xylem	Growing in moist matrix	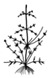
*Metacladophyton* [21,22]	Frasnian	Dichotomous with paired tips curving	Adventitious roots	On rhizome	On stems, 1st and 2nd branches	Rhizome with roots, giving rise to monopodial aerial branching system, with a height of ca. 1 m	Unknown	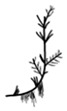
*Shougangia* [3,23]	Famennian	Laminate	Adventitious roots	On one side of procumbent stems	Not observed	Stem prostrating at the portions where roots occur, and then going upright	Unknown	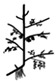
*Rhacophyton* [24,25,26]	Famennian	Planate	Adventitious roots	On stems and bases of sterile fronds, with occasional bifurcation	Not observed	Scrambling shrub adept at clonal propagation possessing long fronds that could reiterate when touching the substrate	Living in palaeoequatorial costal swamps and fluvial back swamps	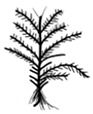
*Melvillipteris* [27]	Famennian	Alternate and dichotomous with paired opposite curving tips	Adventitious roots	On one side of stems’ internodes	Not observed	Herbaceous plant with thin stems with a procumbent and/or upright habit	Living in moist matrix	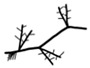
*Ellesmeris* [28]	Frasnian	Laminate	Adventitious roots	On one side of stems and primary pinna	Not observed	Stems upright or horizontal, with a capacity for reiteration	Living in moist substrate	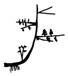
*Protopteridophyton* [29]	Givetian to Frasnian	Dichotomizing for several times and unlaminate	Adventitious roots	On rhizome	Not observed	Herbaceous, annual, with a height of 0.5 m; horizontal rhizome subterranean with aerial branching system upright	Unknown	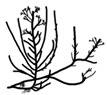
*Flabellopteris* [30]	Famennian	Dichotomizing isotomously	No roots found		Not observed	1st axes upright and connected to a horizontal rhizome	Unknown	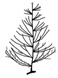
Iridopteridaleans [31,32]	Middle to Late Devonian	Dichotomous and recurved	No roots found		Spines on the whole body in most taxa	(Pseudo)monopodial habit with upright stems up to several centimeters wide	Unknown	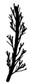

1st = primary, 2nd = secondary.

**Table 2:** Comparisons of Xinhang forest with other Devonian forests or tree assemblages.

Forests/Assemblages [References]	Age	Locality	Composition	Environment
Lindlar assemblage [42,43]	Eifelian	Rhineland, Germany	pseudosporochnaleans (*Calamophyton*, *Weylandia*, *Hyenia*), aneurophytaleans, herbaceous lycopsid	Living near marine
Gilboa forest [11,18]	Givetian	New York, USA	pseudosporochnaleans (*Eospermatopteris*), aneurophytaleans	Wetland coastal plain, limited in duration and subject to periodic disturbance
Cairo forest [12]	Givetian	New York, USA	pseudosporochnaleans (*Eospermatopteris*), *Archaeopteris*, lycopsids	Distal floodplain system in a subtropical to temperate wetland environment, soils well-drained with periodic wet/dry seasonality
Svalbard forest [13]	Frasnian	Svalbard, Norway	lycopsids (*Protolepidodendropsis*)	Tropical, localized, rapidly subsiding, short-lived basin
Xinhang forest [14,15]	Famennian	Anhui, China	lycopsids (*Guangdedendron*), fern-like plants	Costal environment with frequent flood
*Archaeopteris* assemblages/forests [2,26,46]	Late Devonian to Carboniferous	Worldwide	progymnosperms (*Archaeopteris*), fern-like plants	Poorly drained flood plains, coastal areas, upland ecosystems

## Data Availability

The data presented in this study are available in this article and Supplementary Materials.

## Supplementary Materials

The following supporting information can be downloaded at: https://www.mdpi.com/article/10.3390/life14050602/s1, Figure S1: Interpretative line drawings of fern-like plants in Figure 2, Figure 3 and Figure 4 under the same scale; Figure S2: Fern-like plants and associated lycopsid trees from the Yongchuan mine; Figure S3: Fern-like plants and associated lycopsid trees from the Yongchuan mine.

## References

[1] Capel E., Cleal C.J., Xue J.Z., Monnet C., Servais T., Cascales-Miñana B. (2022). The Silurian–Devonian terrestrial revolution: Diversity patterns and sampling bias of the vascular plant macrofossil record. Earth-Sci. Rev..

[2] Taylor T.N., Taylor E.L., Krings M. (2009). Paleobotany: The Biology and Evolution of Fossil Plants.

[3] Wang D.M., Xu H.H., Xue J.Z., Wang Q., Liu L. (2015). Leaf evolution in early-diverging ferns: Insights from a new fern-like plant from the Late Devonian of China. Ann. Bot..

[4] Champreux A., Meyer-Berthaud B., Decombeix A.L. (2020). *Keraphyton* gen. nov., a new Late Devonian fern-like plant from Australia. Peerj.

[5] Niklas K.J., Crepet W.L. (2020). Morphological (and not anatomical or reproductive) features define early vascular plant phylogenetic relationships. Am. J. Bot..

[6] Durieux T., Lopez M.A., Bronson A.W., Tomescu A.M.F. (2021). A new phylogeny of the cladoxylopsid plexus: Contribution of an early cladoxylopsid from the Lower Devonian (Emsian) of Quebec. Am. J. Bot..

[7] Retallack G.J. (1997). Early Forest Soils and Their Role in Devonian Global Change. Science.

[8] Meyer-Berthaud B., Soria A., Decombeix A.L. (2010). The land plant cover in the Devonian: A reassessment of the evolution of the tree habit. Geol. Soc. Lond. Spec. Publ..

[9] Berry C.M. (2019). Palaeobotany: The Rise of the Earth’s Early Forests. Curr. Biol..

[10] Pawlik Ł., Buma B., Šamonil P., Kvačel J., Gałązka A., Kohout P., Malik I. (2020). Impact of trees and forests on the Devonian landscape and weathering processes with implications to the global Earth’s system properties—A critical review. Earth-Sci. Rev..

[11] Stein W.E., Berry C.M., Hernick L.V., Mannolini F. (2012). Surprisingly complex community discovered in the mid-Devonian fossil forest at Gilboa. Nature.

[12] Stein W.E., Berry C.M., Morris J.L., Hernick V.A., Mannolini F., Straeten C.V., Landing E., Marhall J.E.A., Wellman C.H., Beerling D.J. (2020). Mid-Devonian *Archaeopteris* roots signal revolutionary change in earliest fossil forests. Curr. Biol..

[13] Berry C.M., Marshall J.E.A. (2015). Lycopsid forests in the early Late Devonian paleoequatorial zone of Svalbard. Geology.

[14] Wang D.M., Qin M., Liu L., Liu L., Zhou Y., Zhang Y.Y., Huang P., Xue J.Z., Zhang S.H., Meng M.C. (2019). The most extensive Devonian fossil forest with small lycopsid trees bearing the earliest stigmarian roots. Curr. Biol..

[15] Gao X., Liu L., Qin M., Zhou Y., Mao L., Wang D.M. (2022). Re-study of *Guangdedendron micrum* from the Late Devonian Xinhang forest. BMC Ecol. Evol..

[16] Yang J.N., Wang D.M. (2022). A new fern-like plant *Xinhangia spina* gen. et sp. nov. from the Upper Devonian of China. Biology.

[17] Xu P., Liu L., Wang D.M. (2022). Reinvestigation of the Late Devonian Lycopsid *Sublepidodendron grabaui* from Anhui Province, South China. Biology.

[18] Stein W.E., Berry C.M., Hernick L.V., Mannolini F. (2021). The classic mid-Devonian *Eospermatopteris* localities, Gilboa NY, USA. Rev. Palaeobot. Palynol..

[19] Davies N.S., McMahon W.J., Berry C.M. (2024). Earth’s earliest forest: Fossilized trees and vegetation-induced sedimentary structures from the Middle Devonian (Eifelian) Hangman Sandstone Formation, Somerset and Devon, SW England. J. Geol. Soc..

[20] Xue J.Z., Hao S.G. (2008). *Denglongia hubeiensis* gen. et sp. nov., a new plant attributed to Cladoxylopsida from the Upper Devonian (Frasnian) of South China. Int. J. Plant Sci..

[21] Wang D.M., Lin Y.J. (2007). A new species of *Metacladophyton* from the Late Devonian of China. Int. J. Plant Sci..

[22] Wang Z., Geng B.Y. (1997). A new Middle Devonian plant: *Metacladophyton tetraxylum* gen. et sp. nov. Palaeontogr. Abt. B Palaeophytol..

[23] Wang D.M., Zhang Y.Y., Liu L., Xu H.H., Qin M., Liu L. (2018). Reinvestigation of the Late Devonian *Shougangia bella* and new insights into the evolution of fernlike plants. J. Syst. Palaeontol..

[24] Andrews H.N., Phillips T.L. (1968). *Rhacophyton* from the Upper Devonian of West Virginia. Bot. J. Linn. Soc..

[25] Cornet B., Phillips T.L., Andrews H.N. (1976). The morphology and variation in *Rhacophyton ceratangium* from the Upper Devonian and its bearing on frond evolution. Palaeontogr. Abt. B.

[26] Scheckler S.E. (1986). Geology, Floristics and Paleoecology of Late Devonian Coal Swamps from Appalachian Laurentia (U.A.A.). Ann. Société Géologique Belg..

[27] Xue J.Z., Basinger J. (2016). *Melvillipteris quadriseriata* gen. et sp. nov., a new plant assigned to Rhacophytales from the Upper Devonian (Famennian) of Arctic Canada. Geol. Mag..

[28] Hill S.A., Scheckler S.E., Basinger J.F. (1997). *Ellesmeris sphenopteroides* gen. et sp. nov., a new zygopterid fern from the Upper Devonian (Frasnian) of Ellesmere, N.W. T., Arctic Canada. Am. J. Bot..

[29] Li C.S., Hsü J. (1987). Studies on a new Devonian plant *Protopteridophyton devonicum* assigned to primitive fern from South China. Palaeontogr. Abt. B.

[30] Gess R.W., Prestianni C. (2022). *Flabellopteris lococannensis* gen. et sp. nov.: A new fern-like plant from the Famennian of South Africa. Rev. Palaeobot. Palynol..

[31] Berry C.M., Stein W.E. (2000). A new iridopteridalean from the Devonian of Venezuela. Int. J. Plant Sci..

[32] Cordi J., Stein W.E. (2005). The Anatomy of *Rotoxylon dawsonii* comb. nov. (Cladoxylon dawsonii) from the Upper Devonian of New York State. Int. J. Plant Sci..

[33] Soria A., Meyer-Berthaud B., Scheckler S.E. (2001). Reconstructing the architecture and growth habit of *Pietzschia levis* sp. nov. from the Late Devonian of southeastern Morocco. Int. J. Plant Sci..

[34] Berry C.M., Edwards D. (1996). *Anapaulia moodyi* gen. et sp. nov.: A probable iridopteridalean compression fossil from the Devonian of western Venezuela. Rev. Palaeobot. Palynol..

[35] Skog J.E., Banks H.P. (1973). *Ibyka amphikoma* gen. et sp. nov., a new protoarticulate precursor from the late Middle Devonian of New York State. Am. J. Bot..

[36] Wang Y., Berry C.M. (2001). A new plant from the Xichong Formation (Middle Devonian), South China. Rev. Palaeobot. Palynol..

[37] Rayner R.J. (1983). New observations on Sawdonia ornate from Scotland. Trans. R. Soc. Edinb. Earth Sci..

[38] Liu L., Wang D.M., Meng M.C., Xue J.Z. (2017). Further study of Late Devonian seed plant *Cosmosperma polyloba*: Its reconstruction and evolutionary significance. BMC Evol. Biol..

[39] Dunn M.T., Krings M., Mapes G., Rothwell G.W., Mapes R.H., Keqin S. (2003). *Medullosa steinii* sp. nov., a seed fern vine from the Upper Mississippian. Rev. Palaeobot. Palynol..

[40] Hanley M.E., Lamont B.B., Fairbanks M.M., Rafferty C.M. (2007). Plant structural traits and their role in anti-herbivore defence. Perspect. Plant Ecol. Evol. Syst..

[41] Meyer-Berthaud B., Decombeix A.L., Girard C., Steemans P., Blanchard R., Champreux A., Evreïnoff M. (2021). A Late Devonian plant assemblage from New South Wales, Australia: Diversity and specificity. Rev. Palaeobot. Palynol..

[42] Giesen P., Berry C.M. (2013). Reconstruction and growth of the early tree *Calamophyton* (Pseudosporochnales, Cladoxylopsida) based on exceptionally complete specimens from Lindlar, Germany (Mid-Devonian): Organic connection of *Calamophyton* branches and *Duisbergia* trunks. Int. J. Plant Sci..

[43] Giesen P., Berry C.M. (2015). Der älteste Wald der Welt-die einzigartige mitteldevonische Pflanzenfundstelle Lindlar. Archäologie Im Rheinl..

[44] Pigg K.B. (1992). Evolution of Isoetalean Lycopsids. Ann. Mo. Bot. Gard..

[45] Gastaldo R.A., Stevanovic-Walls I.M., Ware W.N., Greb S.F. (2004). Community heterogeneity of Early Pennsylvanian peat mires. Geol. Soc. Am..

[46] Greb S.F., DiMichele W.A., Gastaldo R.A. (2006). Evolution and importance of wetlands in earth history. Spec. Pap. Geol. Soc. Am..

[47] Cross A.T., Phillips T.L. (1990). Coal-forming plants through time in North America. Int. J. Coal Geol..

[48] Cressler W.L. (2006). Plant paleoecology of the Late Devonian Red Hill locality, northcentral Pennsylvania, an Archaeopteris-dominated wetland plant community and early tetrapod site. Spec. Pap. Geol. Soc. Am..

[49] Cressler W.L., Daeschler E.B., Slingerland R., Peterson D.A., Vecoli M., Clement G., MeyerBerthaud B. (2010). Terrestrialization in the Late Devonian: A palaeoecological overview of the Red Hill site, Pennsylvania, USA. Terrestrialization Process: Modelling Complex Interactions at the Biosphere-Geosphere Interface.

[50] Scotese C.R. (2016). PALEOMAP PaleoAtlas for GPlates and the PaleoData Plotter Program, PALEOMAP Project. http://www.earthbyte.org/paleomap-paleoatlas-forgplates/.

[51] DiMichele W.A., Phillips T.L. (2002). The ecology of Paleozoic ferns. Rev. Palaeobot. Palynol..

