# Supplementary material for: Fern-like Plants Establishing the Understory of the Late Devonian Xinhang Lycopsid Forest
Source: Life (Basel). 2024 May 8;14(5):602. doi: 10.3390/life14050602 (PMC11121898; doi:10.3390/life14050602)
Supplement: Supplementary file 1 [file life-14-00602-s001.zip › life-2948057-supplementary.pdf]

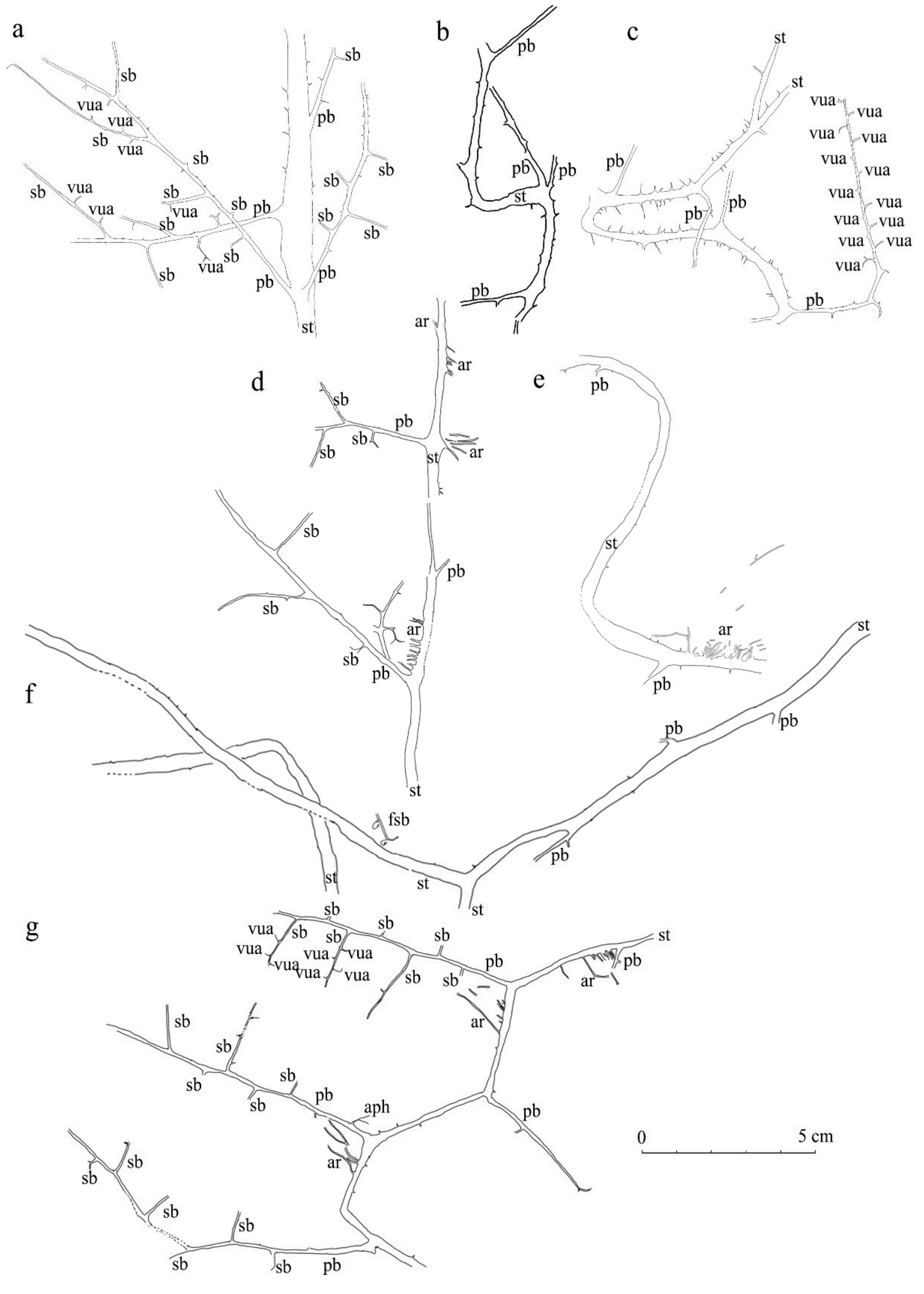

Figure S1. Interpretative line drawings of fern-like plants in Figures 2-4 under the same scale. Source of each picture: **a**: Figure 2a; **b**: Figure 2b; **c**: Figure 2e; **d**: Figure 3a, arrows 1, 2; **e**: Figure 3b; **f**: Figure 3c; **g**: Figure 4a, b (combination of part and counterpart). **Abbreviation**: st: stem; pb: primary branch; sb: secondary branch; fsb: fertile secondary branch; vua: vegetative ultimate appendage; ar: adventitious root; aph: aphlebia.

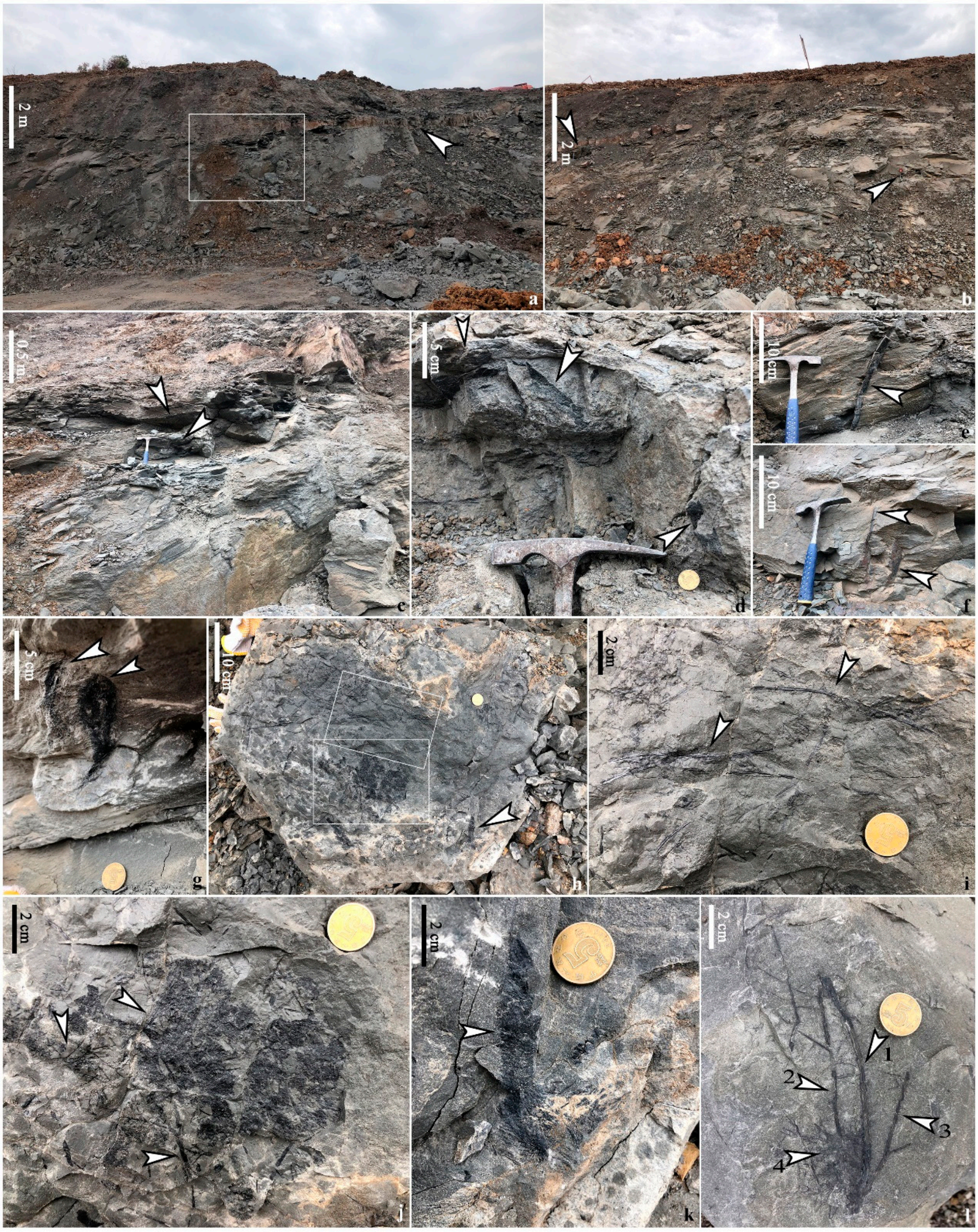

Figure S2. Fern-like plants and associated lycopsid trees from the Yongchuan mine. (**a, b**) The lowest strata of Leigutai Member in the Yongchuan mine. The rectangular box and arrow in **a** indicating parts enlarged in **c** and **d**, respectively. Arrowed parts in **b** from left to right enlarged in **e**, **f**, respectively. (**c**) Enlargement of rectangular part in **a**. The area (lower arrow) near the hammer enlarged in **g**. (**d**) Enlargement of arrowed part in **a**. Two in-situ stems (middle and right arrows) and one horizontally preserved stem (left arrow) of *Guangdedendron micrum*. (**e, f**) Enlargement the arrowed parts in **b**, showing *in situ* stems of *Guangdedendron micrum*. (**g**) Enlargement of arrowed area (lower arrow) in **c**, indicating strobili (arrows) perpendicular to bedding plane. (**h**) Fallen block from the arrowed position (upper arrow) in **c**, showing fern-like plants and strobilus preserved together. The areas in upper and lower rectangles enlarged in **i** and **j**, respectively. The arrowed part is enlarged in **k**. (**i, j**) Enlarged parts in rectangular

boxes in **h**, indicating fragments of fern-like plants (arrows). (**k**) Arrowed part in **h**, indicating strobilus preserved along the bedding (arrow). (**l**) Fallen block from the position near the hammer in **c**, showing stems of fern-like plants (arrows 1-3) with possible adventitious roots (arrow 4).

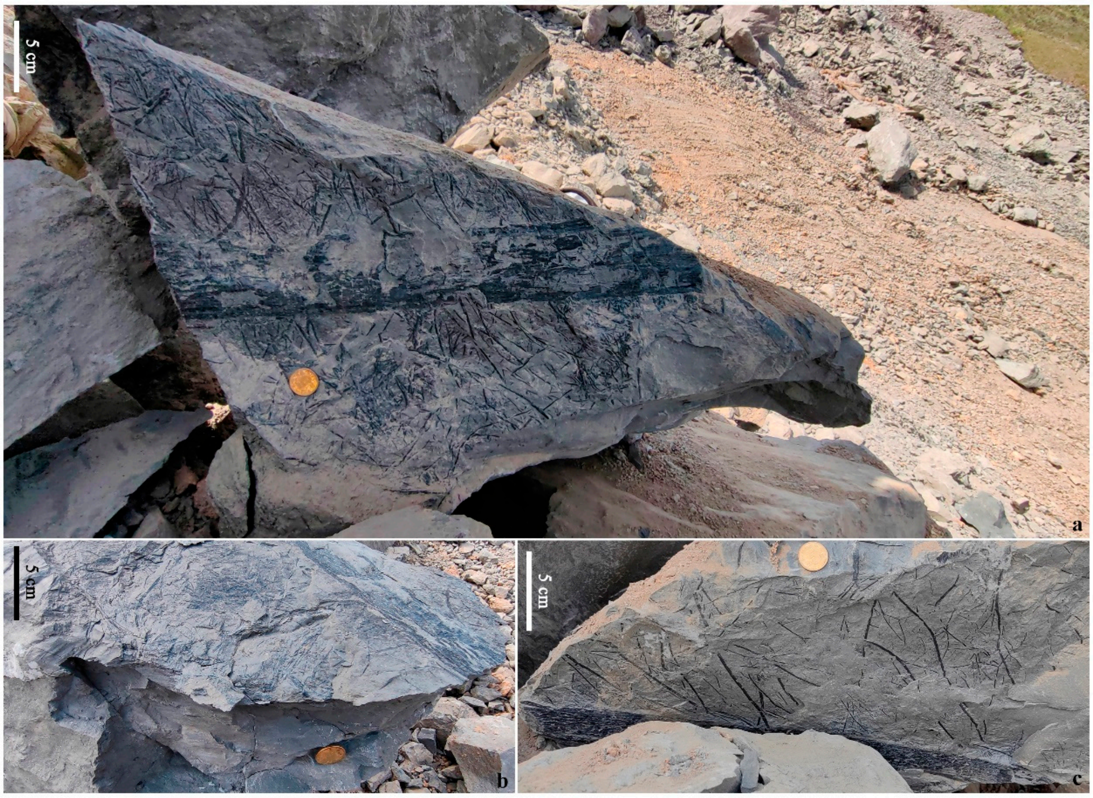

Figure S3. Fern-like plants and associated lycopsid trees from the Yongchuan mine. (**a**, **b**) Part and counterpart, showing fern-like plants associated with a stem of *Guangdedendron micrum* along a bedding plane. (**c**) Fern-like plants and stem of *Guangdedendron micrum* along a bedding plane.
